# Role of histone post-translational modifications in preeclampsia: from pathogenesis to diagnostic and therapeutic strategies

**DOI:** 10.3389/fendo.2026.1973542

**Published:** 2026-10-12

**Authors:** Yuanxuan Ma, Renwei Zhang, Mengting Yang, Jingli Wang, Bo Hou, Shiguo Liu

**Affiliations:** 1 Prenatal Diagnosis Center, The Affiliated Hospital of Qingdao University, Qingdao, Shandong, China; 2 Department of Medical Genetics, The Affiliated Hospital of Qingdao University, Qingdao, Shandong, China; 3 Department of Laboratory, Peking University People’s Hospital Qingdao Hospital, Qingdao, Shandong, China; 4 Department of Cardiology, The Affiliated Hospital of Qingdao University, Qingdao, Shandong, China

**Keywords:** acetylation, histone post-translational modifications, methylation, placenta, preeclampsia

## Abstract

Preeclampsia (PE) is a pregnancy-specific hypertensive disorder that poses a severe risk to maternal and fetal health. Owing to its incompletely elucidated pathogenesis, there is a lack of etiology-targeted therapeutic approaches in clinical practice, and pregnancy termination remains the primary intervention for PE. Emerging evidence indicates that histone post-translational modifications (HPTMs), a crucial class of epigenetic regulatory mechanisms, are extensively involved in the key pathological processes of PE by dynamically remodeling chromatin structures and regulating gene transcription. These processes include trophoblast dysfunction, placental vascular homeostasis dysregulation, immune-inflammatory disturbances, and endothelial dysfunction. In this review, we summarized the regulatory roles of HPTMs, such as methylation, acetylation, lactylation, citrullination, and ubiquitination, in PE pathogenesis. In addition, we discussed the potential and limitations of HPTMs and key modifying enzymes as candidate therapeutic targets and diagnostic biomarkers of PE. Overall, this review aims to summarize the reported roles of HPTMs in PE pathogenesis and examine promising diagnostic and therapeutic strategies from HPTMs perspective.

## 1 Introduction

Preeclampsia (PE), a multisystem inflammatory disorder characterized by the onset of hypertension accompanied by proteinuria or organ dysfunction after 20 weeks of gestation, is a leading cause of maternal and perinatal mortality (1). PE is clinically stratified into early-onset preeclampsia (EOPE), manifesting before 34 weeks of gestation and typically associated with impaired spiral artery remodeling and severe fetal growth restriction, and late-onset preeclampsia (LOPE), occurring at or after 34 weeks, which often presents with milder placental pathology and predominant maternal endothelial involvement (2). Notably, the global prevalence of PE ranges from 2% to 6%, accounting for over 70,000 maternal deaths and more than 500,000 fetal and neonatal deaths annually (3, 4). In addition, PE is associated with an elevated long-term risk of cardiovascular disease, renal disease, and stroke in affected women. Moreover, neonates face multiple health challenges attributable to prematurity and fetal growth restriction (5, 6). Despite several studies, the pathogenesis of PE remains unknown. Given its heterogeneous clinical presentation, effective methods for early management and etiology-specific treatments are lacking. Low-dose aspirin initiated at 12–16 weeks of gestation is considered potentially effective in reducing the incidence of preeclampsia in high-risk women (7). However, inconsistent findings have been reported in a randomized controlled trial conducted among Chinese populations (8). Currently, placental delivery via pregnancy termination is the only definitive intervention to halt disease progression (9). Therefore, a deeper understanding of the molecular mechanisms underlying PE is urgently needed to develop effective preventive and therapeutic strategies.

Epigenetics is defined as the study of reversible and heritable changes in gene expression that occur without alterations in DNA sequence (10). Notably, the major areas of epigenetic regulation include histone modifications, DNA methylation, and non-coding RNA mechanisms. Collectively, these processes dynamically modulate chromatin structure and accessibility, thereby precisely controlling transcriptional activity (11–13). Histone post-translational modifications (HPTMs) encompass several reversible covalent chemical alterations, including acetylation, methylation, and phosphorylation (14). Importantly, these modifications are tightly regulated by “writer” and “eraser” enzymes and often interact with other epigenetic layers, playing a widespread role in the pathophysiology of various diseases, including PE.

High-throughput techniques, such as chromatin immunoprecipitation sequencing, have facilitated the exploration of aberrant HPTMs landscapes in the placentas of patients. Previous studies have shown that the dysregulation of multiple HPTMs is associated with the development of PE. For instance, abnormal enrichment of histone H3 lysine 9 trimethylation (H3K9me3) at the promoter regions of placental angiogenesis-related genes leads to their transcriptional repression. In contrast, dysregulated expression of histone H3 lysine 9 acetylation (H3K9ac) in genes that govern trophoblast invasion impairs trophoblast infiltration capacity (15, 16). Collectively, these findings provide novel insights into the epigenetic pathogenesis of PE. However, the regulatory mechanisms of the HPTMs reported to participate in the development of PE remain incompletely characterized, with their clinical translational potential not yet thoroughly evaluated. Given these gaps, we systematically summarized the characteristics, regulatory roles, and underlying molecular mechanisms of HPTMs in PE, with the aim of providing insights for the development of novel therapeutic strategies and diagnostic approaches for the disease from an epigenetic perspective.

## 2 HPTMs

### 2.1 Chromatin structure and histone composition

Chromatin is a complex structure formed by genetic material and proteins within the nucleus of eukaryotic cells and serves as the fundamental functional form of DNA (17). The basic structural and functional unit of chromatin is the nucleosome, which consists of approximately 147 bp of DNA wrapped around a histone octamer core. Notably, the octamer comprises two copies of each of the four canonical core histones (H2A, H2B, H3, and H4), which form a compact octameric core through interactions between their globular domains, with their N-terminal tails (rich in basic amino acids) extending outward from the nucleosome core particles (18–20). In particular, these tails are key sites for various post-translational modifications and serve as interfaces for interactions with adjacent nucleosomes, regulating higher-order chromatin structures (21–23). Overall, the high conservation of histones combined with the modifiability of their N-terminal tails constitutes the molecular basis for dynamic regulation and functional plasticity of chromatin.

### 2.2 Biological functions of HPTMs

Specific amino acid residues within the N-terminal tails of histones undergo various reversible post-translational modifications (24). Since Allfrey et al. first identified histone acetylation and methylation in 1964, additional modifications, including phosphorylation, ubiquitination, succinylation, and citrullination, have been progressively elucidated, gradually establishing themselves as representative mechanisms of HPTMs (25–27). In recent years, nearly 30 novel types of HPTMs have been discovered and characterized owing to advances in high-resolution mass spectrometry and the development of highly specific modification-specific antibodies. Examples of these novel HPTMs include serotonylation (28), lactylation (29), dopaminylation (30), and histaminylation (31), which markedly expand the chemical diversity of epigenetic regulatory landscapes.

HPTMs play crucial roles in cellular functions and responses to environmental changes by dynamically controlling gene activation or silencing (32). These modifications are dynamically regulated by “writer” and “eraser” enzymes and exert their effects through two principal mechanisms: (1) Altering the net charge of histones via covalent attachment of specific chemical groups or small protein moieties, thereby modifying chromatin architecture. For instance, acetylation and lactylation neutralize positive charges, relaxing chromatin to facilitate transcription (33). Whereas methylation can either relax or condense chromatin, depending on the modified residue and the degree of modification (34, 35). (2) Recruiting downstream effector protein complexes, such as “reader” proteins, including histone protein 1 and polycomb repressive complex. These proteins promote chromatin condensation to maintain a repressive environment or recruit transcriptional coactivators to stabilize the activated state (36–38).

Research has demonstrated that aberrant HPTMs are closely associated with the development and progression of various diseases, including cancer, cardiovascular diseases, neurological disorders, and autoimmune diseases (39–42). Notably, both the placental tissues and peripheral blood of patients with PE exhibit dysregulation of HPTMs profiles, suggesting that the aberrant regulation of HPTMs and their associated enzymes may contribute to the onset and progression of PE (**Table 1**).

**Table 1:** Expression profiles of HPTMs in PE.

Category	Name	Expression level	Localization		Ref.
			Tissue/cell localization	Subcellular localization	
Histone methylation	H3K4me2	↑	Placenta (CTBs, STBs, EVTs)	—	(69)
	H3K4me3	↓	Placenta (STBs), decidua	Nucleus	(89, 93, 190)
	H3K9me1	↑	Placenta (EVTs)	—	(191)
	H3K9me2	↑	Placenta (EVTs, vascular endothelial cells)	Nucleus	(191–193)
	H3K9me3	↑	Placenta (EVTs, vascular endothelial cells)	—	(106, 191, 193)
	H3K27me2	↑	Placenta (EVTs)	Nucleus	(192)
	H3K27me3	↓	Placenta (Hofbauer cells), decidua	—	(106, 107, 194)
	H3R2me2s	↑	Placenta (STBs)	—	(76)
	H4R3me2s	↑	Placenta (STBs)	—	(76)
Histone acetylation	H3K9ac	↓	Placenta (EVTs), decidua	Nucleus	(93, 190)
	H3K27ac	↓	Placenta	—	(131)
Histone lactylation	H3K18la	↑	Placenta	—	(148, 150)
Histone citrullination	CitH3	Placenta↑, serum↓	Placenta (intervillous space), decidua, serum	—	(163, 194, 195)
Histone ubiquitination	H2BK120ub	↑	Placenta (EVTs)	—	(155, 156)
Histone methyltransferase	SETD1A	↑	Placenta (CTVs and STBs)	Nucleus	(69)
	SMYD3	↑	Placenta (CTBs and STBs)	Nucleus, cytoplasm	(69)
	MLL1	↑	Chorionic villi (CTBs)	Nucleus	(89)
	SETD7	↑	Placenta	—	(104, 196)
	G9a	↓	Placenta (vascular endothelial cells)	—	(81)
	EZH2	↓	Placenta	—	(111)
Histone demethylase	KDM5C	↓	Placenta	—	(197)
	KDM3B	↓	Placenta (trophoblast cells)	—	(114)
	JHDM1D	↓	Placenta (EVTs)	Nucleus	(192)
	JMJD1A	↓	Placenta (trophoblast cells in the proximal column and vascular endothelial cells)		(78, 198)
	JMJD2C	↓	Placenta		(79)
	JMJD3	↑	Peripheral blood (PBMCs)		(110)
	JMJD6	Placenta↑ placenta-derived sEVs↓	Placenta (STBs and CTBs), circulating placental small extracellular vesicles	Nucleus	(167, 199, 200)
Histone deacetylase	HDAC1	↓	Blood vessels (subcutaneous adipose tissue)	Nucleus	(144)
	HDAC2	↓	Placenta	Nucleus	(131, 132)
	HDAC3	↓	Blood vessels (subcutaneous adipose tissue)	Nucleus	(126, 144)
	HDAC4	↓	Placenta (trophoblast cells)	Nucleus	(134, 138, 201)
	HDAC5	↓	Placenta (trophoblast cells)	—	(129)
	HDAC6	↓	Placenta, blood vessels (subcutaneous adipose tissue)	Cytoplasm	(124, 144)
	HDAC8	↓	Placenta	—	(126)
	HDAC9	↓	Placenta (STBs)	—	(126, 135, 202)
	HDAC10	↓	Placenta	—	(126)
	SIRT1	↓	Placenta (STBs, CTBs), serum	Cytoplasm	(170, 203)
	SIRT2	↓	Placenta (STBs, CTBs, vascular endothelial cells)	Nucleus, cytoplasm	(128, 203)

## 3 HPTMs in the pathogenesis of PE

In normal pregnancy, extravillous trophoblasts (EVTs) invade the uterine decidua and spiral artery walls, displacing the original vascular smooth muscle layer in concert with fibrinoid deposition and inducing endothelial cell apoptosis, thereby accomplishing physiological spiral artery remodeling (43). This transformation converts high-resistance spiral arteries into low-resistance, high-capacitance vessels that ensure adequate uteroplacental blood flow to meet the nutrient and oxygen demands of the developing fetus (44, 45). However, the invasiveness of EVTs is compromised in patients with PE, resulting in superficial remodeling of the spiral artery and consequent placental ischemia–hypoxia, which induces oxidative stress and endoplasmic reticulum stress, releasing inflammatory mediators and antiangiogenic factors into the maternal circulation and precipitating systemic endothelial dysfunction and vascular inflammation (46–50). Although the specific molecular cascade underlying PE is unclear, defective spiral artery remodeling and resultant placental maladaptation are widely regarded as the primary origins of its pathophysiology. This pathological process is associated with multiple dysregulated molecular mechanisms, including aberrant epithelial–mesenchymal transition (EMT) of trophoblasts (51), disordered hypoxic responses (52), maternal–fetal immune imbalance at the decidual interface (53), excessive activation of inflammatory signaling pathways (54), and dysregulation of pyroptosis and autophagy (55, 56). Emerging evidence indicates that epigenetic modulation, particularly HPTMs, extensively influences the cellular and biological processes implicated in PE pathogenesis (**Figure 1**). In this review, we summarize the current advances in the regulatory roles of various HPTMs in impaired spiral artery remodeling and the subsequent pathological sequelae of PE, aiming to provide insights into potential therapeutic targets directed at the underlying mechanisms of PE.

**Figure 1:**
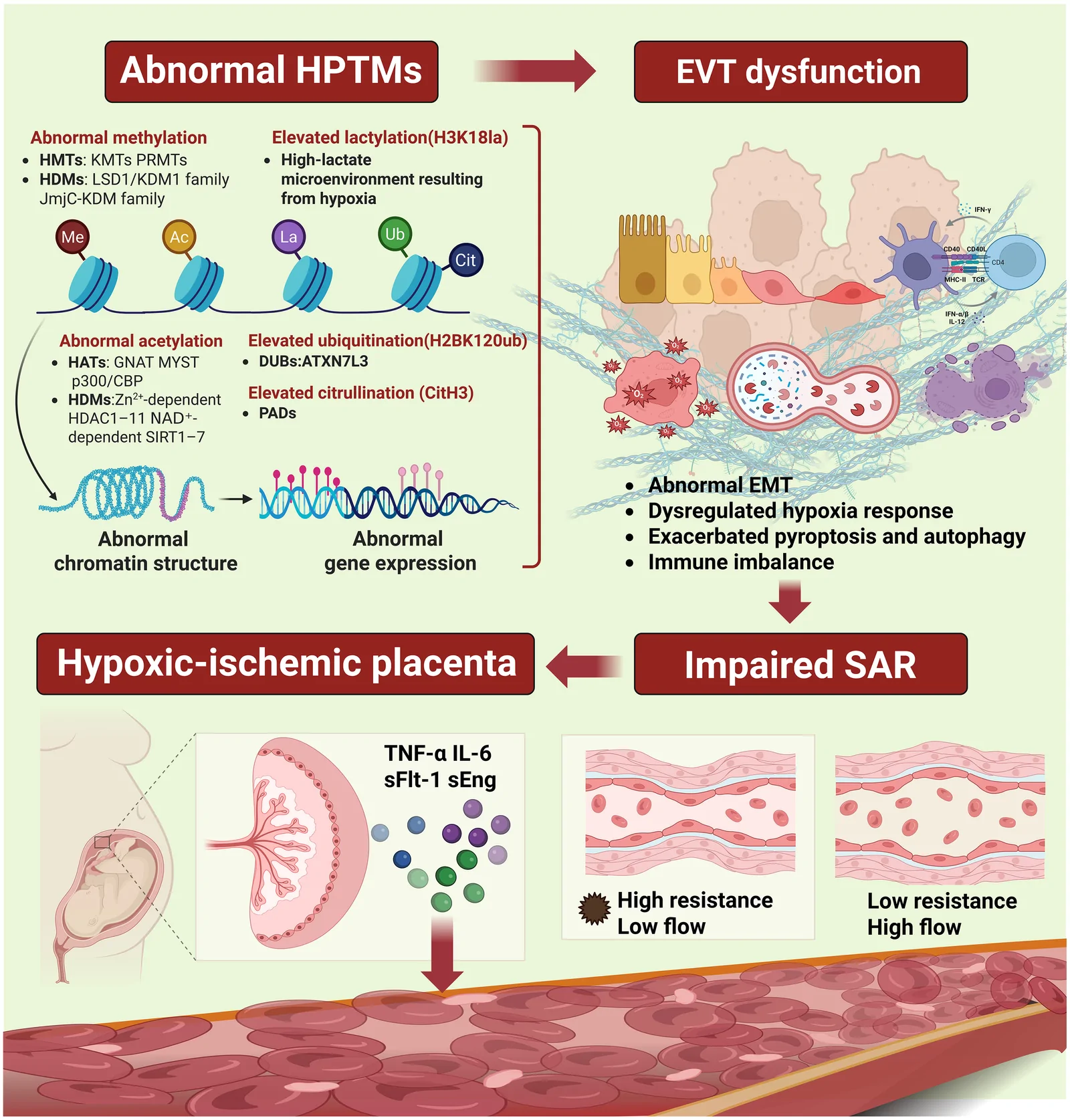
Roles of HPTMs in the pathogenesis of PE. HPTMs (methylation, acetylation, lactylation, ubiquitination, and citrullination) lead to trophoblast dysfunction by abnormally regulating biological processes such as EMT, the hypoxic response, immune homeostasis, pyroptosis, and autophagy. The resulting impairment of spiral artery remodeling subsequently leads to placental ischemia and hypoxia, accompanied by the release of inflammatory mediators and anti-angiogenic factors into the maternal circulation.

### 3.1 Histone methylation

#### 3.1.1 Overview of histone methylation

Histone methylation involves the addition of one or multiple methyl groups (me1/me2/me3) to amino acid residues on histones and is widely distributed across the N-terminal tail regions of core histones, particularly H3 and H4. Lysine and arginine are the predominant modification sites (57, 58). The methylation status is dynamically controlled by histone methyltransferases (HMTs) and demethylases (HDMs) (59). HMTs comprise lysine (KMTs) and arginine methyltransferases based on substrate specificity (60), whereas HDMs include the LSD1/KDM1 and JmjC domain-containing (JmjC-KDM) families for lysine demethylation, and enzymes such as Jumonji domain-containing protein 6 (JMJD6) for arginine demethylation (61–63).

Methylation modifications at distinct histone residues exhibit evolutionarily conserved and functionally specific characteristics. H3K4me, H3K36me, and H3K79me are enriched in the promoters or gene bodies of actively transcribed genes and are associated with transcriptional activation (35, 64, 65). In contrast, H3K9me, H3K27me, and H4K20me are predominantly localized to heterochromatic regions or promoters of silenced genes, exerting transcriptionally repressive effects (66–68). In PE placentas, Alterations in the levels of various histone methylation markers, including H3K27me3 and H3K4me3, profoundly affect the transcriptomic profiles and functional status of trophoblasts (**Figure 2**).

**Figure 2:**
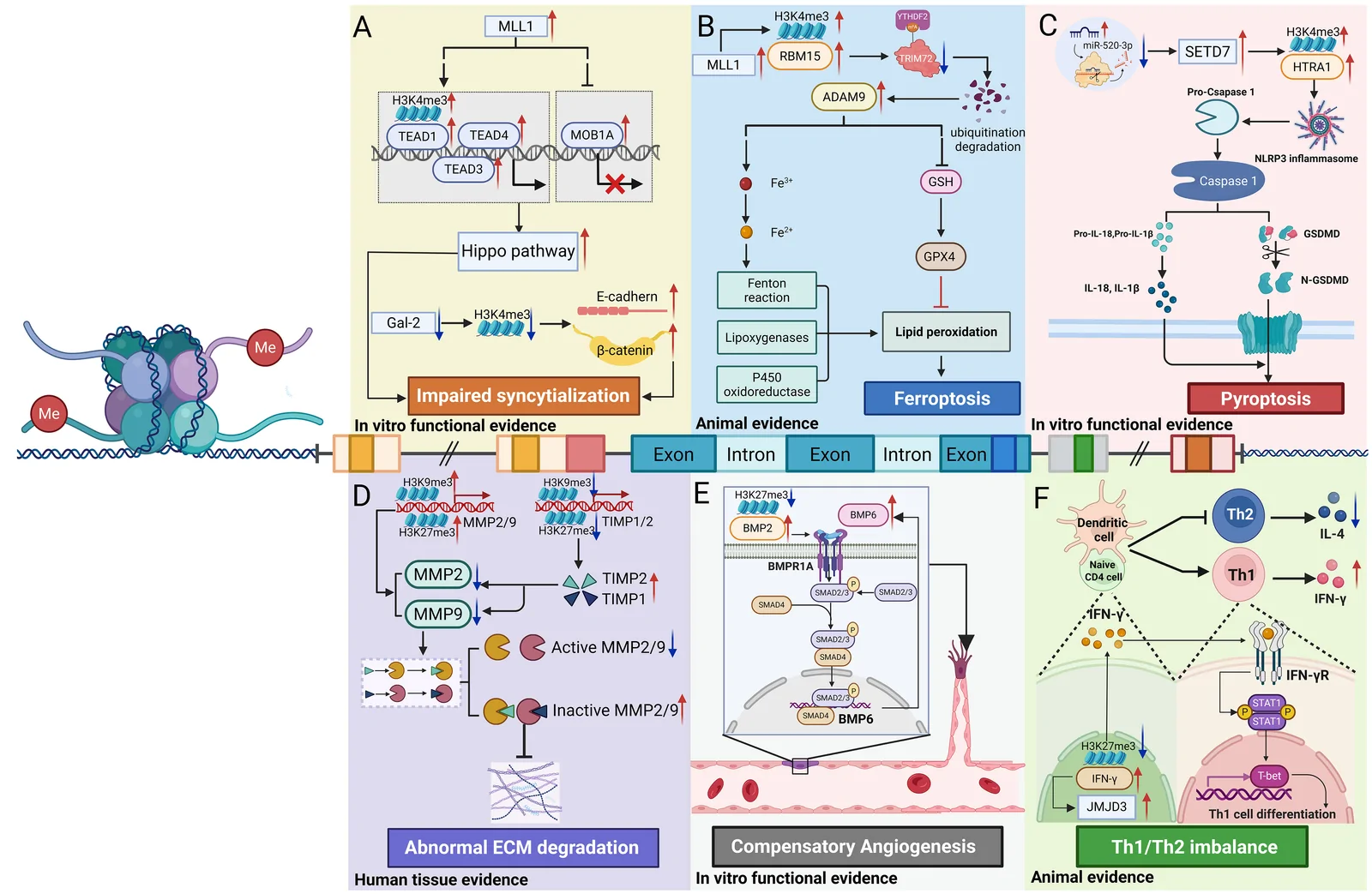
Dysregulated histone methylation in PE placenta​. Dysregulation of HMTs and HDMs in PE placenta drives multiple pathologies via aberrant histone methylation. **(A)** Impaired syncytialization: In CTBs, upregulated MLL1(HMT) catalyzes H3K4me3 to positively regulate TEAD4, activating the Hippo pathway and impairing CTB syncytialization. Reduced H3K4me3 in STBs, mediated by Gal-2 dysregulation, suggests suggest a global transcriptional failure in STBs. **(B)** Trophoblast ferroptosis: Upregulated MLL1 drives H3K4me3–RBM15–m^6^A–YTHDF2–TRIM72–ADAM9 axis, triggering ferroptosis. **(C)** Trophoblast pyroptosis: Overexpressed SETD7(HMT) upregulates HTRA1 via H3K4me3, activates the NLRP3/caspase-1/GSDMD pathway, and thereby induces pyroptosis. **(D)** Abnormal ECM degradation: Upregulation of H3K9me3 and H3K27me3 in the MMP2/MMP9 promoter regions, accompanied by a concomitant decrease in repressive modifications at the TIMP2/TIMP1 loci, disrupts the MMP/TIMP balance, leading to reduced ECM degradation capacity **(E)** Compensatory angiogenesis: Low H3K27me3 in PE placenta promotes BMP2 transcription, activating the BMPR1A–SMAD2/3–SMAD4 cascade to upregulate BMP6 and promote compensatory neovascularization. **(F)** Th1/Th2 imbalance: Upregulated JMJD3(HDM) catalyzes the demethylation of H3K27me3 to lift IFN-γ-mediated transcriptional repression, driving the initial CD4^+^T cells toward a Th1-like phenotype and promoting a pro-inflammatory immune response in PE.

#### 3.1.2 Histone methylation in hypoxic response

Pathological hypoxia resulting from impaired uterine spiral artery remodeling drives the aberrant regulation of histone methylation, which represents a critical mechanism underlying placental injury in PE (**Figure 3**). Matsui et al. demonstrated that the histone methyltransferases SETD1A and SMYD3 are overexpressed in EOPE placentas, particularly in trophoblast cells, compared with both full-term healthy pregnancies and gestational age-matched preterm pregnancies without hypertension. In addition, their co-catalyzed products, H3K4me2 and H3K4me3, are elevated under hypoxic conditions and promote transcriptional activation by enriching the promoter regions, thereby influencing trophoblast functions (69). Another study reported that H3K4me3 is enriched at the promoter of lncRNA-HEIPP, a long non-coding RNA upregulated by hypoxia-inducible factor 1α (HIF-1α) in the placenta of patients with PE, with higher enrichment rates observed after 24 h of hypoxia. Functional assays showed that H3K4me3 suppresses trophoblast invasion by upregulating the expression of lncRNA-HEIPP (70). However, this study did not stratify PE subtypes, and the control group comprised full-term pregnancies, making it impossible to exclude the contribution of gestational age differences to the observed H3K4me3 enrichment. In contrast to previous study, this investigation did not observe hypoxia-induced global changes in H3K4me3 levels. Notably, this discrepancy likely arises from differences in experimental design. Although an earlier study detected increased H3K4me3 levels within 8–16 h of hypoxia, the latter study applied hypoxic treatment for 24, 48, and 72 h in the same HTR-8/SVneo cell line. Overall, these findings suggest time-dependent dynamics of H3K4me3 in response to hypoxic stress. During early hypoxia, HIF-1α mediates a rapid elevation of H3K4me3 to initiate the transcription of hypoxia-adaptive genes. As hypoxia persists (≥ 24 h), the expression of H3K4me3 demethylases may be induced to restore H3K4me3 levels and maintain epigenetic homeostasis, thereby preventing excessive gene activation. At this stage, H3K4me3 is preferentially enriched in the promoters of effector genes to regulate their expression. Future studies should map the genome-wide distribution of H3K4me3 at different hypoxic time points to identify the target genes regulated by this marker at each phase, thereby elucidating the mechanistic role of H3K4me3 in the hypoxic response.

**Figure 3:**
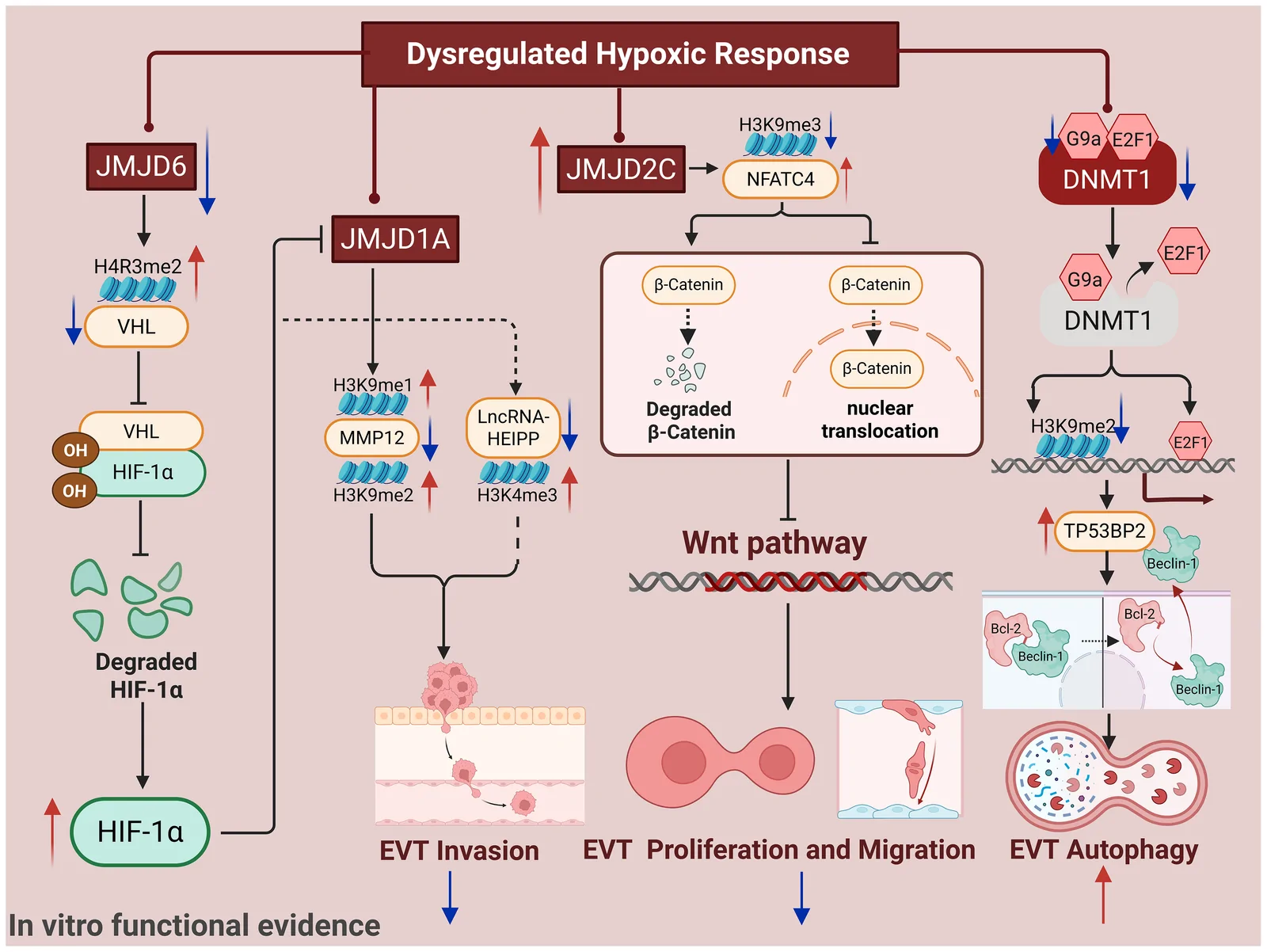
Abnormal histone methylation induced by pathological hypoxia mediates placental injury. Dysregulation of JMJD6, JMJD1A, JMJD2C, and G9a in trophoblasts impairs hypoxic adaptation through distinct pathways. Inactivated JMJD6 induces a high HIF-1α microenvironment in the placenta of PE via the H3R3me2-VHL-HIF-1α axis. Highly expressed HIF-1α impairs the invasive capacity of trophoblast cells by inhibiting the JMJDA1-H3K9me1/H3K9me2-*MMP12* axis and the H3K9me3-LncRNA-HEIPP axis. JMJD2C upregulation suppress invasion and proliferation of trophoblast cells via H3K9me3-*NFATC4-β*-Catenin-Wnt signaling pathway. Downregulated G9a cooperates with DNMT1 to upregulate *TP53BP2* and promote Beclin-1 release, ultimately inducing aberrant trophoblast autophagy.

Under physiological conditions, the hypoxic response of trophoblast cells is maintained by hypoxia-inducible factors (HIFs), whose stability is regulated by oxygen-dependent HIF hydroxylases, including members of the JmjC-KDM family (71, 72). JMJD6 is the only member of the JmjC domain-containing metalloenzyme family capable of removing histone arginine methylation and promoting gene expression through oxygen-dependent demethylation of H3R2me2 and H4R3me2 (73). Von Hippel Lindau (VHL), a regulator of gene expression under hypoxic conditions, plays a critical role in placental hypoxia responses during pregnancy, particularly through its oxygen tension-dependent targeting and ubiquitin-mediated degradation of HIF-1α, thereby maintaining placental angiogenesis homeostasis and syncytiotrophoblasts (STBs) development. Importantly, this oxygen tension dependence is associated with the demethylase activity of JMJD6 (74, 75). Compared with physiological placental hypoxia (8% O_2_), the demethylase activity of JMJD6 toward repressive H4R3me2 at the *VHL* promoter is markedly reduced in JEG3 cells under PE-associated hypoxic conditions (3% O_2_), leading to decreased VHL protein expression. This finding suggests that JMJD6 dysregulation-induced H4R3me2 elevation may amplify or maintain hypoxia-related placental dysfunction. However, this speculation is based on *in vitro* trophoblast cell model rather than direct evidence from PE placental tissues. Therefore, its pathological relevance to PE remains hypothetical and requires further validation. Notably, this decline in JMJD6 demethylase activity was exclusively observed in EOPE and was reversible (76).

Unlike JMJD6, which regulates HIF-1α, JMJD1A, a member of the JmjC-KDMs family, may participates in the hypoxic adaptive response of PE trophoblast cells as a downstream regulator of HIF-1α (71, 72). One of the functions of JMJD1A is to bind to the promoter region of the hypoxia-responsive effector *MMP12*, which significantly reduces the level of the repressive modification H3K9me, thereby promoting *MMP12* expression (71, 77). JMJD1A and *MMP12* are co-localized in trophoblast cells within the proximal column of first-trimester human placental villi, which serve as the source of EVTs. Study based on *in vitro* cell models and rat models demonstrated that under hypoxic conditions (0.5% O_2_ for cell models; 10.5% O_2_ for rat models), low levels of JMJD1A inhibit HIF-induced demethylation of H3K9me1 and H3K9me2, leading to suppressed *MMP12* expression and impaired invasive capacity of hypoxia-activated trophoblasts (78). JMJD2C, another member of the JmjC-KDM family, is upregulated in the placenta of patients with PE and in hypoxia-treated HTR-8/SVneo cells. Although gestational age-matched controls were employed, the study did not stratify the enrolled PE patients by subtype. JMJD2C ameliorates transcriptional repression by reducing H3K9me3 levels in the promoter region of *NFATc4*. In addition, the subsequent upregulation of *NFATc4* inhibits the Wnt/β-catenin signaling pathway by blocking β-catenin nuclear translocation, ultimately suppressing trophoblast proliferation and migration (79).

G9a, a member of HMT, is implicated in hypoxia-induced autophagic overactivation, an important mechanism underlying EOPE-related placental injury. This process involves a coordinated interplay between DNA methylation and histone methylation, leading to the overexpression of tumor protein p53-binding protein 2 (*TP53BP2*) (80, 81). Using *in vitro* trophoblast cell models, Jiang et al. demonstrated that the DNA methyltransferase DNMT1 forms a functional complex with G9a and the transcription factor E2F1. G9a catalyzes the enrichment of H3K9me2 in the TP53BP2 promoter, whereas DNMT1 mediates DNA methylation in the same region, establishing a compact chromatin configuration. This condensed chromatin architecture physically obstructs E2F1 from accessing its direct target, *TP53BP2*. Moreover, the physical interaction between DNMT1 and E2F1 inhibits the transcriptional activation of E2F1. Under hypoxic conditions, the downregulation of DNMT1 and G9a shifts the chromatin to an open state, allowing E2F1 to bind and activate *TP53BP2* transcription. *TP53BP2* overexpression promotes beclin-1 release, enhancing autophagic activity in trophoblasts, consistent with observations in trophoblasts derived from EOPE placentas (81).

#### 3.1.3 Histone methylation in impaired trophoblast syncytialization

After implantation, trophoblasts differentiate into stem cell-like cytotrophoblasts (CTBs), which subsequently give rise to EVTs or fuse via syncytialization to form multinucleated STBs (82). Syncytialization is fundamental to the maintenance of maternal–fetal nutrient and gas exchange and embryonic organ development during pregnancy (83–85). Impaired syncytialization or dysfunction of STBs is a key pathological feature of PE and has been linked to dysregulated histone methylation (86).

Mixed-lineage leukemia 1 (MLL1), a SET domain-containing lysine methyltransferase (KMT) that catalyzes H3K4me3, is a critical epigenetic regulator of trophoblast syncytialization (87–89). Wu et al. demonstrated that MLL1 expression in trophoblasts gradually decreases during syncytialization in normal pregnancy. Compared with gestational age-matched uncomplicated pregnancies, MLL1 and its mediated H3K4me3 are aberrantly upregulated in placental villi from patients with PE in the third trimester, and this upregulation is closely associated with impaired trophoblast fusion. In particular, the direct target gene of MLL1 is TEA domain transcription factor 4 (*TEAD4*), a key regulator of trophoblast self-renewal and stemness maintenance. Alterations in MLL1 expression are associated with placental injury resulting from aberrant trophoblast lineage differentiation or dysfunction (90, 91). Cell-based functional assays in trophoblasts revealed that MLL1 positively regulates *TEAD4* expression by promoting H3K4me3 enrichment in the promoter region, thereby activating the Hippo signaling pathway and inhibiting syncytialization. Notably, this process is modulated by the hypoxic microenvironment, in which hypoxia-inducible factor HIF-1A directly binds to the MLL1 promoter and enhances its transcription. Dysregulation of MLL1, as a potential upstream driver, activates the H3K4me3/TEAD4/Hippo signaling axis. In contrast, Hahn et al. showed that impaired trophoblast fusion in PE is associated with downregulated H3K4me3 levels, the expression of which is positively correlated with patient age. Loss of H3K4me3 is caused by the downregulation of the placental growth factor galectin-2 (Gal-2), suggesting that Gal-2 supplementation may treat PE by upregulating H3K4me3 levels (92, 93).

We speculate that the discrepancy between the conclusions of the two studies may be due to differences in the stages of trophoblast differentiation, despite both studies utilizing the placenta as their subjects. The MLL1/H3K4me3 axis suppresses the differentiation of precursor cells responsible for generating new STBs via the activation of TEAD4, thereby preventing their fusion into STBs. However, Gal-2 is primarily located in terminally differentiated STBs, which require a stable transcriptional program to perform and maintain syncytium formation. In this context, the observed reduction in H3K4me3, a transcriptional activation marker, suggests diminished transcriptional activity and cellular function of STBs in PE, with Gal-2 potentially serving as a supporting signal to maintain the syncytial state. In summary, MLL1-mediated aberrant upregulation of H3K4me3 in specific genes underlies the blockade of CTB syncytialization. In contrast, Gal-2 dysregulation-driven downregulation of H3K4me3 reflects impaired maintenance of the syncytial state due to reduced STB transcriptional activity. In addition, the overall decrease in H3K4me3 levels observed in the whole placental tissue may indicate a state of global transcriptional exhaustion in the placenta of patients with PE.

#### 3.1.4 Histone methylation in trophoblast ferroptosis

In addition to its role in trophoblast syncytialization, MLL1 contributes to the pathogenesis of PE by mediating ferroptosis in trophoblasts (94). Ferroptosis is an oxidative stress-dependent form of programmed cell death characterized by the accumulation of labile iron and reactive oxygen species (ROS), resulting in the generation of toxic lipid peroxidation products (95). In the pathological hypoxic microenvironment of PE, electron microscopy revealed that trophoblast mitochondria in patient-derived samples exhibited typical ferroptotic features, notably reduced membrane density and diminished cristae formation (96). Decreased levels of glutathione (GSH) and glutathione peroxidase 4 (GPx4) in the serum and trophoblasts of patients with PE are important indicators of exacerbated lipid peroxidation (97, 98).

Evidence from functional assays in HTR-8/SVneo cells indicated that MLL1 positively regulates the transcription of RNA-binding motif protein 15 (*RBM15*) by increasing the level of H3K4me3 modifications in the promoter region. RBM15, functioning as an N^6^-methyladenosine (m^6^A) writer, increases m^6^A modification of *TRIM72* and promotes its recognition and degradation by YTHDF2, thereby suppressing TRIM72-mediated ubiquitination and degradation of ADAM9 and enhancing ADAM9 stability (94). Consequently, intracellular levels of Fe²^+^, malondialdehyde, and ROS are elevated, accompanied by decreased GSH levels and GPx4 inactivation, triggering lipid peroxidation and ferroptosis in trophoblast cells (99, 100). This mechanism was validated in a mouse PE model established by tail vein injection of sFlt-1 adenovirus. However, given that the research remains at the preclinical stage, whether the MLL1–RBM15–m^6^A–TRIM72–ADAM9 axis acts as an upstream driver of trophoblast ferroptosis in PE awaits further validation in PE patient-derived tissues.

#### 3.1.5 Histone methylation in trophoblast pyroptosis

Pyroptosis is a type of programmed inflammatory cell death that involves the activation of the NLRP3 inflammasome and caspase-mediated cleavage of gasdermin D (GSDMD), leading to the formation of plasma membrane pores and the release of pro-inflammatory cytokines, such as IL-1β and IL-18 (101). Pyroptosis is significantly elevated in the placenta and trophoblast cells of patients with PE, and dysregulation of histone methylation is a key upstream mechanism driving this process.

Previous studies have shown that the expression of the serine protease HTRA1 is four times higher in the placentas of women with PE than that in normal placentas. In addition, dysregulated HTRA1 expression is directly associated with pathological alterations in abnormal placental development (102, 103). Compared with healthy pregnant women at the same gestational age, the histone methyltransferase SETD7 is upregulated in the placentas of patients with PE. In H/R-treated trophoblast cell models simulating the PE environment, SETD7 catalyzes H3K4me3 enrichment at the *HTRA1* promoter region, thereby promoting HTRA1 transcription. Aberrantly high *HTRA1* expression activates the NLRP3/caspase-1/GSDMD signaling axis, triggering trophoblast pyroptosis (104).

#### 3.1.6 Histone methylation in extracellular matrix (ECM) degradation

The balance between matrix metalloproteinases (MMPs) and their tissue inhibitors (TIMPs) is essential for effective trophoblast invasion into the maternal decidua, a process involving the degradation of ECM components (105). In PE, this equilibrium is disrupted by aberrant histone methylation. Compared with normal full-term, gestational age-matched placental villi, H3K9me3 and H3K27me3 at the MMP2 and MMP9 promoter regions were observed to be upregulated by 1.7- and 1.8-fold, respectively, in PE villi, leading to suppressed MMP2 and MMP9 expression. Concurrently, the expression of *TIMP1* and *TIMP2* increased because of reduced modification by these two proteins, further inhibiting MMP activity. Importantly, this disruption of the MMP/TIMP balance results in diminished ECM-degrading capacity, which is closely associated with the shallow trophoblast invasion phenotype observed in PE (106). However, this study did not classify the enrolled subjects by PE subtype, necessitating further validation in subtype-defined cohorts.

#### 3.1.7 Histone methylation in placental angiogenesis dysregulation

Although elevated H3K27me3 is a detrimental factor in ECM degradation, it plays a compensatory regulatory role in placental neovascularization in PE through aberrant activation of BMP2 signaling. Compared with full-term healthy pregnant women, global H3K27me3 levels are reduced in the placentas of patients with PE, including both EOPE and LOPE, and this alteration affects key components of the BMP signaling pathway. Evidence from *in vitro* cell experiments indicated a reduction in H3K27me3 in the *BMP2* promoter region leads to increased *BMP2* expression, which in turn upregulates *BMP6* expression via the BMPR1A-SMAD2/3-SMAD4 signaling pathway. As an invasion-promoting factor, *BMP2* enhances the invasive capacity of trophoblast cells by activating the transcription of angiogenesis-related genes such as placental growth factor (PlGF) and MMPs. In an Flt1-induced rat PE model, BMP2 supplementation alleviated the hypertensive and fetal growth restriction phenotypes. BMP6 is an indispensable intermediary molecule in *BMP2*-meDIATED regulation of trophoblast invasion and angiogenesis (107). In addition, the expression of several important angiogenic factors in the placenta of patients with PE is regulated by histone trimethylation. A previous study revealed that although H3K9me3 levels were elevated in the VEGF promoter, H3K27me3 exhibited aberrant enrichment in EGFR and c-Jun promoters. Notably, this repressive methylation significantly downregulated the transcriptional levels of VEGF, EGFR, and c-Jun, thereby compromising the proper formation of the placental vascular network (15).

#### 3.1.8 Histone methylation in immune imbalance

Helper T (Th) cell-mediated immune dysregulation is a critical component of the pathogenesis of PE. This is characterized by excessive activation of Th1-type immune responses (exemplified by pro-inflammatory cytokines such as IFN-γ) and suppression of Th2-type responses (marked by anti-inflammatory cytokines such as IL-4), resulting in a shift in the Th1/Th2 ratio toward a pro-inflammatory direction (108, 109). Importantly, this immune imbalance exacerbates local placental and systemic inflammatory responses, ultimately contributing to the onset and progression of PE. Previous studies have demonstrated that the histone demethylase JMJD3 plays a regulatory role in this process through epigenetic mechanisms. Mechanistically, JMJD3 differentially modulates H3K27me3 modifications at the promoter regions of *IFN-γ* and *IL-4*, thereby influencing Th1/Th2 cell differentiation. In addition, JMJD3 catalyzes the demethylation of H3K27me3 at the *IFN-γ* promoter, relieving transcriptional repression and promoting IFN-γ expression. In contrast, JMJD3 enhances H3K27me3 methylation at the *IL-4* promoter, thereby suppressing IL-4 transcription. Overall, this differential regulation by JMJD3 drives naïve CD4+ T cells toward aberrant Th1 polarization, thereby disrupting the Th1/Th2 balance (110). However, since the study was based on a mouse PE model, the impact of the proposed mechanism on the immune microenvironment in patients with PE remains unclear. The expression patterns and functional relevance of the JMJD3–H3K27me3 axis in placental tissues and peripheral blood immune cells from PE patients require validation in clinical samples. Collectively, these findings suggest that targeting the JMJD3–H3K27me3 regulatory axis may be a potential therapeutic strategy for restoring maternal–fetal immune homeostasis.

#### 3.1.9 Histone methylation in pathways associated with trophoblast dysfunction

In HTR-8/SVneo and JEG3 trophoblast cell lines, reduced expression of enhancer of zeste homolog 2 (EZH2) attenuates its catalytic activity toward H3K27me3 and H3K4me2, thereby relieving the transcriptional suppression of genes such as *GADD45A*, *ANGPTL4*, and *MT2A*. This leads to aberrant activation of signaling pathways, including p38/MAPK, impairing trophoblast proliferation and migration (111–113).

Guo et al. detected reduced expression of peroxisome proliferator-activated receptor gamma (PPARγ) in PE placentas and H/R-treated trophoblasts. *In vitro* experiments revealed that PPARγ knockdown attenuated its effect on KDM3B-mediated demethylation of H3K9me2 at *Activated leukocyte cell adhesion molecule* (*ALCAM*) promoter, thereby inhibiting the Wnt/β-catenin pathway and impairing trophoblast function (114). Collectively, these findings suggest that histone-modifying enzymes may serve as critical upstream epigenetic nodes regulating trophoblast function and that restoration of their activity or expression may represent a promising therapeutic strategy for delaying or treating PE.

### 3.2 Histone acetylation

#### 3.2.1 Overview of histone acetylation

Histone acetylation involves the addition of an acetyl group to lysine residues, which neutralizes positive charges and promotes a more open chromatin conformation to activate transcription (115–119). The homeostasis of this process is coordinated by histone acetyltransferases (HATs) and deacetylases (HDACs) (120). HATs comprise three major families (GNAT, MYST, and p300/CBP) that promote gene expression (121–123), whereas HDACs remove acetyl groups to restore chromatin compaction and transcriptional repression. In humans, 18 HDACs have been identified, including zinc-dependent HDAC1–11 and NAD-dependent SIRT1–7 (124). HDACs are generally downregulated in the placenta of patients with PE and are closely associated with the onset and progression of PE (**Figure 4****) (**124–128). For example, HDAC5 and HDAC6 downregulation impairs trophoblast function by persistently activating the CD31/mTOR pathway or inhibiting vascular endothelial growth factor A (VEGFA) (129, 130).

**Figure 4:**
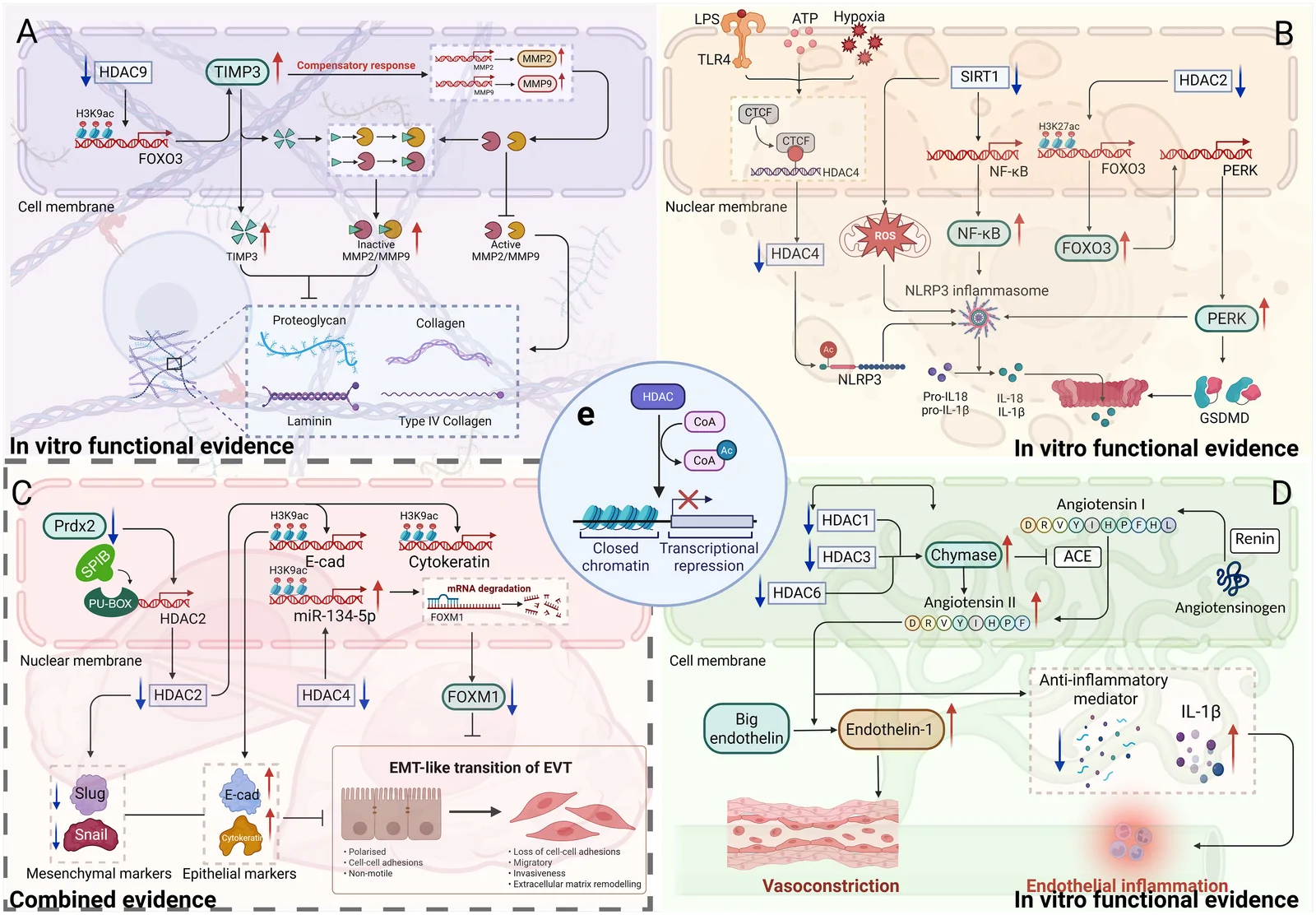
Regulatory mechanisms of downregulated HDACs in PE. HDACs catalyze the hydrolysis of acetyl groups on lysine residues at the N-terminal tail of histones, which restores the positive charge of the histones and promotes chromatin condensation with subsequent transcriptional repression. In PE placentas, widespread inactivation of HDACs contributes to impaired spiral artery remodeling and vascular dysfunction through the following pathways: **(A)** Downregulation of HDAC9 impairs ECM degradation. **(B)** Reduced expression of HDAC2, HDAC4, and SIRT1 triggers excessive trophoblast pyroptosis. **(C)** Downregulation of HDAC2 and HDAC4 inhibits EMT in trophoblast cells (Hypothetical evidence derived from studies on recurrent miscarriage and cancers). **(D)** Coordinated downregulation of HDAC1, HDAC3, and HDAC6 mediates vasoconstriction, endothelial inflammation, and increased vascular permeability. **(E)** Schematic diagram of HDAC-mediated gene transcription repression.

#### 3.2.2 Histone acetylation in trophoblast pyroptosis

In addition to histone methylation, trophoblast pyroptosis in the placenta of patients with PE is associated with histone hyperacetylation. HDAC2, HDAC4, and SIRT1 exhibit anti-pyroptotic activity in trophoblasts through different mechanisms. HDAC2 indirectly affects pyroptotic pathways by regulating histone acetylation. HDAC2 downregulation in PE placental tissues upregulates H3K27ac levels in the promoter region of the transcription factor *FOXO3*, thereby activating transcription. *FOXO3* overexpression upregulates PERK, an endoplasmic reticulum stress-associated kinase, which promotes NLRP3 inflammasome activation, caspase-1 cleavage, and GSDMD-N pore formation, ultimately enhancing the secretion of IL-1β and IL-18 (131). However, the absence of gestational age information for both case and control groups, together with the lack of PE subtype stratification, precludes excluding the influence of gestational age differences on H3K27ac levels and FOXO3 expression and leaves the contributions of this mechanism to EOPE and LOPE pathogenesis undetermined. Moreover, HDAC2 downregulation facilitates the activation of the NF-κB pathway, thereby amplifying upstream signals for NLRP3 inflammasome activation and further promoting pyroptosis (132). Similarly, in LPS-induced mouse PE models and trophoblast cell models, SIRT1 inhibition promotes NLRP3 activation and IL-1β secretion via the NF-κB pathway (133).

Unlike HDAC2 and SIRT1, HDAC4 exerts anti-pyroptotic effects through direct deacetylation of key pyroptotic proteins. Under hypoxic conditions or in PE-like pathological states simulated by LPS/ATP, the accumulation of the transcriptional repressor CTCF in the *HDAC4* promoter region increases, leading to the downregulation of HDAC4 expression. HDAC4 downregulation reduces the ability to deacetylate NLRP3 and GSDMD, thereby promoting NLRP3 inflammasome assembly and GSDMD pore formation, ultimately exacerbating pyroptosis (134). However, as described above, studies on the impact of histone deacetylase dysregulation on trophoblast pyroptosis are largely based on *in vitro* cell experiments or animal experiments that simulate the pathological state of PE, with direct evidence from PE patient placental tissues being limited.

#### 3.2.3 Histone acetylation in ECM degradation

In contrast to the relatively straightforward regulatory pattern of histone methylation, the regulation of matrix metalloproteinases and their inhibitors by histone acetylation exhibits a more complex compensatory balance and spatial specificity. In pre-eclamptic placentas, the H3 hyperacetylation state resulting from HDAC9 downregulation simultaneously upregulates *TIMP3* expression and enhances the intracellular activity of MMP2/MMP9. The latter may represent a compensatory stress response in trophoblasts with elevated *TIMP3* expression (135). However, MMPs primarily degrade ECM components as secreted or membrane-anchored proteins, suggesting that intracellular MMP activity is insufficient to determine ECM degradation efficiency (136). Secreted TIMP3 is anchored in the ECM, forming an effective barrier in the extracellular microenvironment that keeps functional MMPs secreted into the extracellular space in an inhibited state. Consequently, increased intracellular MMP activity may not alter the pathological phenotype of impaired trophoblast invasion following HDAC9 knockdown. However, this study was limited to a preliminary investigation of the potential mechanisms of HDAC9 in PE development, leaving its role in subtype-specific PE pathology unaddressed.

#### 3.2.4 Histone acetylation in EMT impairment

The migratory and invasive capacities of EVTs depend on EMT, which is characterized by the downregulation of the epithelial marker E-cadherin and upregulation of mesenchymal markers. In PE, the EMT process is generally attenuated and is closely associated with abnormal histone acetylation (16). Studies in trophoblast cell models have shown that peroxiredoxin 2 (Prdx2) induces the transcription factor SPIB to recognize the PU-box element within the *HDAC2* promoter, thereby activating *HDAC2* transcription. Activated HDAC2 subsequently binds to the E-cadherin promoter, suppressing *E-cadherin* transcription by reducing H3K9 acetylation levels and upregulating the expression of the mesenchymal markers Slug and Snail, thus driving EMT (137). However, this study was a mechanistic investigation of recurrent miscarriage. Although the Prdx2–SPIB–HDAC2–E-cad axis regulates trophoblast EMT, there is currently a lack of direct evidence from PE patients to confirm whether abnormalities in this axis directly lead to impaired trophoblast EMT in PE. Therefore, the pathological relevance of this mechanism in PE remains hypothetical and requires further validation in PE-specific models and clinical samples.

In contrast to HDAC2, which directly modifies key EMT genes, HDAC4 indirectly influences EMT by regulating miRNA expression. HDAC4 inhibits the transcriptional expression of miR-134-5p by catalyzing the deacetylation of H3K9 in the miR-134-5p promoter region. miR-134-5p downregulation inhibits the binding of the transcription factor FOXM1. As a key regulator of cancer EMT, FOXM1 upregulation contributes to the acquisition of migratory and invasive phenotypes by trophoblast cells (138–140). However, HDAC4 downregulation disrupts this regulatory axis in PE, potentially impeding EMT. Although the mechanisms remain distinct, both histone deacetylases play a role in regulating EMT and jointly maintain the normal function of trophoblast cells. Overall, these findings provide a theoretical basis for the development of multitarget combination therapy strategies targeting EMT abnormalities in PE. In addition to participating in EMT regulation, low levels of H3K9ac have been shown to contribute to syncytiogenesis defects in the trophoblasts of patients with PE (92, 141).

#### 3.2.5 Histone acetylation in endothelial dysfunction

Angiotensin-converting enzyme (ACE) and angiotensin II (Ang II) are important components of the renin-angiotensin system, and their expression is significantly upregulated in the placental vasculature of patients with PE (142, 143). Studies have revealed a potential balance between ACE and chymase, which coordinately regulate Ang II levels in endothelial cells, and the maintenance of this balance is regulated by histone acetylation. In endothelial cells overexpressing chymotrypsin, feedback inhibition of ACE expression shifts the dominant pathway of Ang II generation from ACE-dependent to chymase-dependent pathway. A previous study revealed that treatment with HDAC inhibitors upregulated chymotrypsin expression in a dose-dependent manner and downregulated ACE expression, suggesting that high histone acetylation promotes chymotrypsin transcription and inhibits ACE expression. Analysis of clinical samples further demonstrated that HDAC1, HDAC3, and HDAC6 levels were downregulated in the subcutaneous adipose tissue of patients with PE, which correlated with elevated chymase levels in the local vasculature (144). However, given that the case and control groups were not gestational age-matched and patients were not stratified by PE subtype, the potential influence of gestational age differences on HDAC1, HDAC3, and HDAC6 expression levels cannot be excluded. Therefore, targeting histone acetylation regulatory pathways may offer novel interventional strategies for ameliorating chymase-mediated endothelial dysfunction.

### 3.3 Other types of HPTMs

#### 3.3.1 Histone lactylation

Lactate, the end product of glycolysis, has been increasingly recognized for its role in energy metabolism, immune regulation, and inflammation (145–147). In 2019, Zhang et al. identified that lactate serves as the substrate for a novel HPTM, histone lactylation, which involves the addition of a lactyl group to lysine residues and, similar to acetylation, promotes transcriptional activation by relaxing chromatin structure (29).

Placental hypoxia increases lactate accumulation, which induces histone lactylation, primarily of H3K18la, thereby exacerbating placental dysfunction. H3K18la, a histone lactylation marker commonly studied in oncology, is elevated in the placenta of patients with PE and contributes to PE pathogenesis through multiple pathways. Importantly, the specific mechanisms through which H3K18la participates in PE were first elucidated in 2021. Under hypoxic conditions, lactate upregulates the fibrosis-related genes *FN1* and *SERPINE1* by promoting H3K18la enrichment in their promoter regions, representing a key pathological pathway that drives placental villous stromal fibrosis in PE (148). Shen et al. demonstrated that H3K18la is involved in the regulation of cuproptosis in trophoblasts. Under hypoxic conditions, extensive H3K18la enrichment activates *GRHL2* expression. *GRHL2*, a transcription factor, binds directly to the promoter region of the copper transporter *SLC31A1*, enhancing its transcriptional activity. This leads to the accumulation of Cu ions, triggering trophoblast coproptosis and suppressing trophoblast proliferation and invasion (149). Recently, premature senescence observed in hypoxic PE placentas has been linked to histone lactylation. Elevated H3K18la levels in the placenta of patients with PE activate the transcription of growth arrest and DNA damage-inducible alpha (*GADD45A*), subsequently inducing trophoblast senescence, as evidenced by increased levels of senescence-associated β-galactosidase (SA-β-Gal) and P16 (150).

#### 3.3.2 Histone ubiquitination

Protein ubiquitination involves the covalent conjugation of ubiquitin to lysine residues on substrate proteins, regulating their function, localization, and stability (151). Histones are among the most heavily monoubiquitinated proteins in mammalian cell nuclei, with this modification primarily occurring at specific lysine sites in H2A and H2B (152). Mono-ubiquitination of H2B at lysine 120 (H2BK120ub) is the most prevalent form and, by inhibiting nucleosomal array folding and interfiber oligomerization, promotes an open chromatin state that facilitates transcriptional activation (153, 154).

Based on an LPS-induced PE trophoblast model, Zhang et al. revealed that H2Bub1 serves as a critical link between NLRP3-mediated trophoblast pyroptosis and glucose metabolic reprogramming. This mechanism involves the abnormal enrichment of H2Bub1 in the promoter region of *TLR4*, which promotes *TLR4* transcription, thereby continuously activating the downstream TLR4–NF-κB–PFKFB3 signaling pathway (155, 156). In addition, excessive activation of this pathway upregulates the protein levels of NLRP3, caspase-1-p10, and GSDMD, and drives the excessive production of IL-1β and IL-18, thereby disrupting inflammatory homeostasis. Moreover, the TLR4–NF-κB–PFKFB3 signaling pathway induces metabolic reprogramming, characterized by enhanced glycolysis and impaired oxidative phosphorylation via upregulation of PFKFB3, a key glycolytic regulatory enzyme. In this process, downregulation of the deubiquitinating enzyme ATXN7L3 is responsible for the abnormal elevation of H2Bub1 levels. This vicious cycle of inflammation and metabolic dysregulation collectively leads to trophoblast dysfunction and may provide new insights into the pathogenesis of PE. Future studies should first examine the expression levels of H2Bub1, ATXN7L3, TLR4, and PFKFB3 in PE patient placental tissues and compare them with those in gestational age-matched normal pregnancy controls. On this basis, the correlation of these indicators with clinical severity and PE subtypes should be further analyzed to clarify the status of this axis under PE pathological conditions. Construction of an *ATXN7L3* knockout mouse model is necessary to verify whether the ATXN7L3–H2Bub1–TLR4 axis drives trophoblast pyroptosis and metabolic reprogramming *in vivo*, and to assess its effects on placental development and function.

#### 3.3.3 Histone citrullination

Histone citrullination is catalyzed by the peptidylarginine deiminase (PAD) family and is dependent on intracellular calcium ion concentration (157, 158). This modification chydrolyzes arginine residues to citrulline and is involved in the onset and progression of diseases such as cancer and rheumatoid arthritis (27, 159, 160).

In the pathophysiology of PE, inflammatory mediators in the placental microenvironment, such as IL-8 and placenta-derived particles, stimulate neutrophil activation, leading to abnormal production and release of NETs (161). Citrullinated histone H3 (CitH3) infiltration is observed in the intervillous space of placental tissues from patients with PE, whereas it is absent in placentas from healthy pregnancies (162). Further studies have revealed that circulating CitH3 levels in patients with EOPE are significantly lower than those in gestational age-matched controls and patients with LOPE; moreover, its expression levels are positively correlated with PlGF and fetal birth weight, and negatively correlated with sFlt-1 in patients with PE (163). These correlations suggest that aberrant CitH3 expression may contribute to the pathological processes underlying abnormal placental development and dysfunction. However, there is currently a lack of functional experimental evidence to support the role of CitH3 in PE, and the mechanism linking it to the clinical phenotype of PE remains to be further elucidated.

## 4 HPTMs: potential therapeutic targets for PE

Previous studies on HPTM profiles, the activity of regulatory enzymes, and transcriptional regulation of downstream genes in PE have advanced the development of novel therapeutic targets. However, research on HPTM-targeted therapies for PE remains in the exploratory stage, with existing studies primarily focusing on interventions targeting histone methylation. In contrast, targeted strategies for other types of modifications are extremely limited (**Table 2**). Although inhibitors or activators of certain key enzymes in HPTM have shown potential for improving placental pathological damage in animal models, the clinical translation of these epigenetic drugs faces challenges.

**Table 2:** Clinical applications of HPTMs in the management of PE.

Therapeutic drugs	Molecular target & mechanism category	Evidence level	Administration route	Dose	Expression profile in PE	Therapeutic effect		Pregnancy safety assessment	Ref.
						Maternal effect	Fetal/placental effect		
MI-3454	Molecular target: MLL1inhibitor; Mechanism categoryz: Direct targeting of a histone-modifying enzyme	Animal evidence: Mouse	Intraperitoneal injection	15 mg/kg	—	—	Increased syncytium formation; increased fetal weight and litter size;improved fetal vascular development	No pregnancy safety assessment	(89)
Hinokitiol	Molecular target: JMJD6 agonist; Mechanism categoryz: Indirect modulation of HPTM-related pathways	Animal evidence: Rat, zebrafish	Intravenous injection	10mg/kg	—	SBP reduced by 30 mmHg within 30 min; Cardioprotection;	—	Cardiovascular and developmental toxicity at high doses	(204)
Metformin	Molecular target: SIRT1 agonist; Mechanism categoryz: Broader pleiotropic effects	RCT evidence: EOPE patients(n=180)	Oral	3000 mg/day	—	Gestational prolongation of approximately 7.6 days	—	Associated with reduced fetal weight and increased risk of IUGR in animal models	(169, 171)
P4	Molecular target: SIRT1 agonist; Mechanism categoryz: Broader pleiotropic effects	RCT evidence: Study 1: Healthy pregnant women (n = 5267) Study 2: PE patients(Vaginal gel/Vaginal micronized progesterone +oral:i.m.+oral= 114:95)	Study 1:Vaginal microcapsules; Study 2: Vaginal gel/Vaginal microcapsules +oral; IM+oral	Study 1: 400 mg bid; Study 2: Vaginal gel+oral:90mg/day+10mg bid; Vaginal microcapsules +oral:200mg/day+10mg bid; IM+oral:60mg qd+10mg bid	↓	Study 1: Early-pregnancy vaginal P4 reduced PE risk (RR 0.61, 95% CI 0.41–0.92, 3 RCTs, I² = 0%, moderate-quality evidence); Study 2: PE incidence significantly lower in the IM+oral vs. Vaginal gel/Vaginal microcapsules +oral (2.97% vs. 4.23%, P = 0.009)	—	More favorable fetal safety profile than metformin	(170, 172, 173)
RESV	Molecular target: SIRT1 agonist; Mechanism categoryz:broader pleiotropic effects	RCT evidence: EOPE patients(NIFE+RESV: NIFE+placebo+174:175)	Oral	50 mg per dose, administered every 15 minutes for up to 5 doses, until blood pressure ≤ 150/100 mmHg	—	Shorter time to blood pressure target attainment, delayed onset of hypertensive crisis, and reduced required NIFE dose compared with NIFE + placebo group	No neonatal adverse events observed	No serious maternal or neonatal adverse events reported in PE patients	(174, 175)
Melatonin	Molecular target: SETD7 inhibitor; Mechanism categoryz:broader pleiotropic effects	Human observational evidence:EOPE patients (n =20)	Oral	30mg/day	↓	Mean interval from diagnosis to delivery prolonged by 6 ± 2.3 days; reduced requirement for antihypertensive agents	No neonatal adverse events observed	No neonatal adverse events observed in a small PE cohort	(176, 177)

SBP, Systolic Blood Pressure; IM, Intramuscular (injection); P4, Progesterone; RESV, Resveratrol; NIFE, Nifedipine; RCT, Randomized Controlled Trial.

### 4.1 MI-3454

H3K4 methyltransferase MLL1 inhibitors have demonstrated potential for the treatment of MLL-rearranged leukemia and solid tumors by effectively blocking the MENIN-MLL1 interaction, thereby specifically downregulating oncogenes (164). Among these, MI-3454, a sub-nanomolar inhibitor developed in 2020, exhibited potent anti-leukemic activity as a single agent in patient-derived xenograft (PDX) models of MLL leukemia (165, 166).

Recent studies have revealed the potential of MI-3454 for treating PE. In the BeWo and JEG3 models, MI-3454 treatment significantly promoted syncytium formation, as evidenced by an increased proportion of multinucleated cells, dose-dependent downregulation of MLL1/H3K4me3 levels, and upregulation of trophoblast syncytium markers. In a mouse model, MI-3454 administration(15mg/kg/day) enhanced placental syncytial layer thickening and fetal vascular development and significantly improved fetal weights and litter sizes. Collectively, these findings provide preclinical evidence supporting the use of MI-3454 to ameliorate syncytialization defects in patients with PE (89). Importantly, its high MLL1 inhibitory activity and oral bioavailability make MI-3454 a valuable candidate drug for improving pathological placental damage in patients with PE. However, rigorous preclinical and clinical evaluations of its safety during pregnancy are required before it can be clinically applied in pregnant patients.

### 4.2 Hinokitiol

High-dose Fe²^+^ supplementation restores the demethylase activity of JMJD6 in EOPE placentas, thereby improving the physiological responses of the placenta to hypoxia (76). However, dietary iron supplementation does not significantly improve the clinical outcomes of PE, which may be attributed to disturbances in iron absorption, transport, and local iron metabolism in the placenta, indicating the need to develop targeted strategies to regulate placental iron homeostasis.

Hinokitiol, a natural iron ionophore, has been shown to restore JMJD6 function by promoting intracellular Fe²^+^ utilization (76, 167). Although direct evidence for its ability to ameliorate placental hypoxic injury remains insufficient, it exerts acute antihypertensive and cardioprotective effects, offering a potential avenue for alleviating PE-related maternal hypertension. Animal experiments have demonstrated that intravenous administration of 10 mg/kg hinokitiol reduces systolic blood pressure by 30 mmHg within 30 min in hypertensive rats without inducing reflex tachycardia or disturbing electrolyte balance.

However, Hinokitiol exhibits dose-dependent cardiovascular and developmental toxicity, which may involve mitochondrial dysfunction and oxidative stress resulting from iron chelation (168). Overall, these findings suggest that although the iron-carrying function of hinokitiol helps restore JMJD6 activity, systemic administration may disrupt maternal-fetal iron homeostasis and pose unpredictable risks to fetal development. Therefore, its safety margin and potential risks must be systematically evaluated in animal models of pregnancy before considering its use in the treatment of PE to ensure that placental function is restored without compromising maternal and fetal safety.

### 4.3 Metformin

Owing to its high safety profile and low cost, metformin has been identified as a candidate drug for preventing and treating PE. Clinical studies have shown that daily administration of 3000 mg metformin prolongs pregnancy by an average of approximately 7.6 days in patients with EOPE. Although the difference was not statistically significant, it demonstrated a clinically meaningful and positive trend (169).

SIRT1 levels are significantly reduced in the placenta and serum of patients with PE, and this reduction mediates excessive pyroptosis and autophagy in trophoblasts (133, 170). *In vivo* experiments demonstrated that metformin intervention (200 mg/kg) significantly ameliorated the late-gestational hypertensive phenotype in SIRT1^+^/^-^ mice, reducing systolic blood pressure from 117.8 ± 8.311 mmHg to 105.8 ± 7.001 mmHg. Although this treatment lowered maternal blood pressure, it was accompanied by a decrease in fetal weight (vehicle vs. treated: 0.6808 ± 0.08630 g vs. 0.6037 ± 0.1168 g) and an increased risk of intrauterine growth restriction. Therefore, metformin may have limitations in improving perinatal outcomes, warranting careful evaluation of its potential impact on fetal development during clinical application. Currently, two clinical trials (PACTR201608001752102 and NTC01353391) are underway to evaluate the efficacy and safety of metformin in the treatment of EOPE (171). In addition, metformin attenuates lipopolysaccharide (LPS)-induced elevation of H2Bub1 status in trophoblast cells and inhibits the activation of the TLR4/NF-κB/PFKFB3 pathway by reducing H2BK120ub enrichment at the *TLR4* promoter, ultimately restoring cellular metabolic and inflammatory homeostasis. Collectively, these findings highlight the multitarget and multi-pathway placental protective properties of metformin (155).

### 4.4 Progesterone (P4)

Compared with metformin, the cholesterol-derived hormone P4 demonstrated a clear advantage in reducing the risk of PE-related adverse pregnancy outcomes. In the SIRT1^+^/^-^ pregnant mouse model, P4 administration (200 mg/kg) exhibited superior antihypertensive efficacy compared with metformin (P4 vs. metformin: 103.0 ± 7.388 mmHg vs. 105.8 ± 7.001 mmHg). Importantly, P4 significantly increased live fetal weight (0.8290 ± 0.08013 g), whereas metformin reduced fetal weight, suggesting that P4 has a more favorable maternal–fetal safety profile. At the cellular level, P4 promoted the invasive capacity of HTR-8/SVneo trophoblasts to a greater extent than metformin, effectively reversing the invasion defect caused by SIRT1 knockdown and significantly inhibiting apoptosis (170). P4 treatment via vaginal or intramuscular injection during early pregnancy has been shown to reduce the incidence of PE. Targeting SIRT1 with P4 provides new evidence for the exploration of HPTMs in the prevention and treatment of PE (172, 173). Future large-scale, multicenter, prospective, randomized controlled trials should be conducted to evaluate the efficacy and safety of P4 treatment regimens with varying doses, routes of administration, and timings of intervention for the prevention and management of PE.

### 4.5 Resveratrol

Evidence from animal and clinical trials has revealed that the SIRT1 agonist RESV, a polyphenolic molecule derived from fruits, can effectively reduce placental oxidative stress and exert antihypertensive effects in patients with PE (174). RESV serves as a safe and effective adjunctive therapy to oral nifedipine (NIFE) for alleviating hypertensive symptoms in PE. A randomized controlled trial involving 349 patients with PE showed that patients in the NIFE plus RESV group achieved a significantly shorter time to blood pressure target attainment than those in the NIFE plus placebo group, with a marked delay in the onset of new hypertensive crisis. In addition, the required therapeutic dose of NIFE was significantly lower in the NIFE+RESV group. Importantly, no serious maternal or neonatal adverse events associated with RESV administration were observed in patients with PE, suggesting that RESV has a favorable clinical safety profile in pregnant populations (175).

### 4.6 Melatonin

Melatonin (5-methoxy-N-acetyltryptamine) has a favorable biosafety profile and has emerged as a potential adjunctive therapy for PE owing to its pronounced antioxidant, anti-inflammatory, and blood pressure-regulating effects (176). Exogenous melatonin attenuates placental oxidative stress, reduces the release of the antiangiogenic factor sFlt-1, and acts directly on the vascular endothelium to improve its function, highlighting its potential value in the management of PE (177).

Recent studies have shown that melatonin suppresses trophoblast pyroptosis by altering histone methylation. Melatonin effectively inhibits the NLRP3 inflammasome-mediated pyroptotic pathway activated by the SETD7/HTRA1 axis through targeted inhibition of *SETD7* expression, a key regulator of pyroptosis in the placenta of patients with PE. Functional assays demonstrated that melatonin treatment effectively inhibited pyroptosis in trophoblasts under hypoxia/reoxygenation (H/R) conditions and enhanced their migratory and invasive capacities (104). Notably, the pharmacological mechanism by which melatonin inhibits trophoblast pyroptosis provides a novel basis for its use as an adjunct therapy for PE, further supporting its potential for clinical translation.

## 5 HPTMs: potential peripheral blood biomarkers for PE

Research on HPTMs in PE has expanded beyond exploring their pathogenic mechanisms to include their clinical value as novel diagnostic biomarker (**Figure 5**). Small extracellular vesicles (sEVs) derived from the placenta play a critical role in the pathological progression of PE and the proteomic information they carry offers a unique window for non-invasive diagnosis. In 2014, researchers profiled the proteome of PE-derived sEVs and identified downregulated histones using 1D Gel-LC-MS/MS. Overall, these findings suggest the presence of DNA repair defects and heightened inflammatory responses in the placenta of patients with PE, providing early clues for leveraging histone information within sEVs for diagnostic purposes (178). However, the accurate detection and analysis of low-abundance histones and specific modifications remain constrained by the limitations of label-free methods and spectral counting employed in early studies.

**Figure 5:**
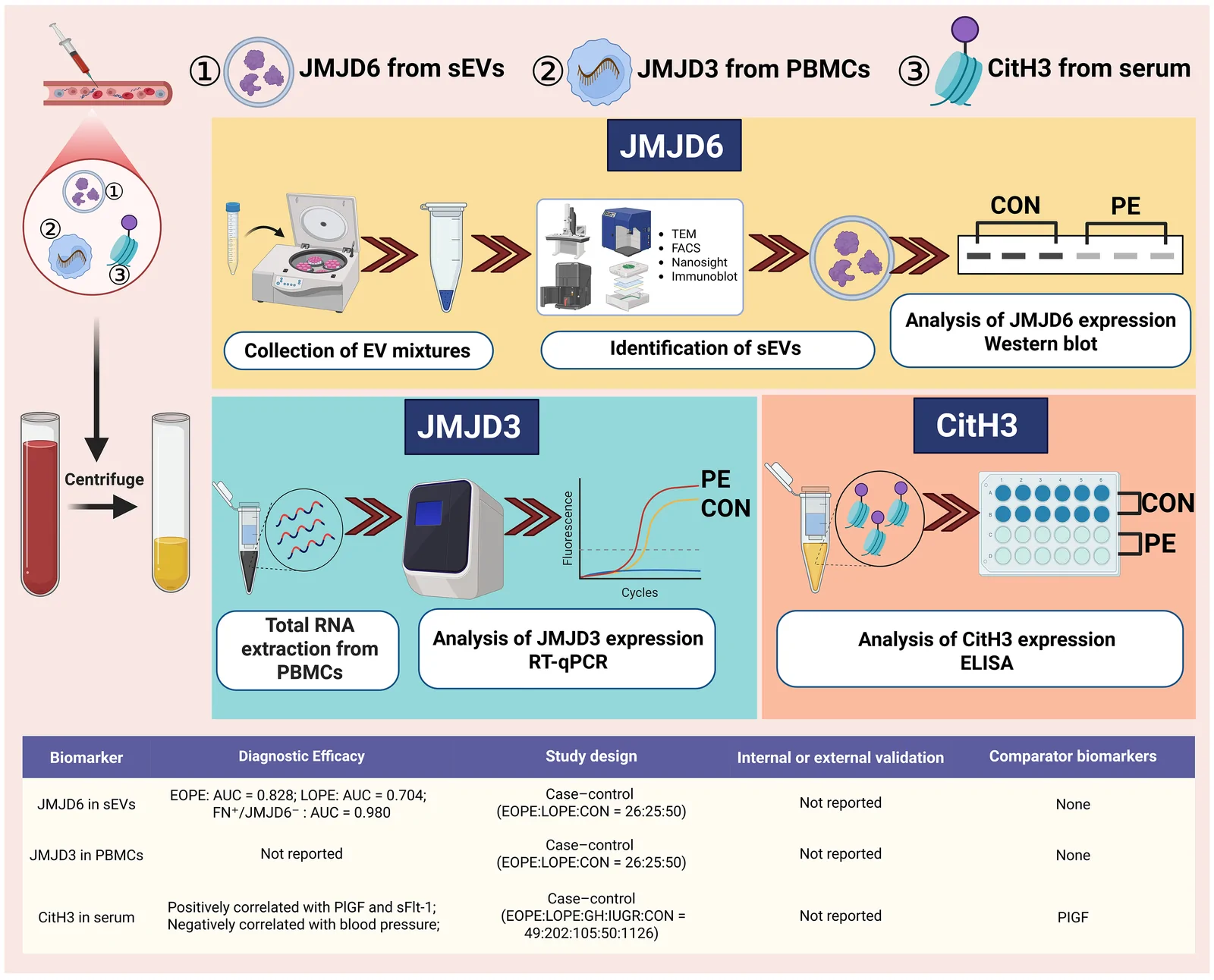
HPTMs as potential peripheral blood biomarkers for PE. JMJD6 in placenta-derived sEVs, PBMCs-derived JMJD3, and serum-derived CitH3 have demonstrated diagnostic potential for PE. This figure illustrated the detection methods and workflows for JMJD6, JMJD3, and CitH3, and summarizes the diagnostic efficacy and translational limitations of each candidate biomarker in tabular form.

Owing to advances in detection technology, researchers have attempted to directly detect histone modifications with greater pathological specificity in the serum. In particular, the development of citrulline detection techniques, such as phenylglyoxal-based probes, has facilitated the visualization of histone citrullination and the discovery of novel epigenetic biomarkers for the disease (179–181). Previous studies have revealed that although CitH3 levels are significantly elevated in the placenta of patients with PE, circulating CitH3 levels are lower than those in healthy pregnant women (163, 182). Importantly, this distribution pattern was positively correlated with PlGF levels. Abnormal PlGF expression promotes the migration of activated neutrophils to the placenta and vascular smooth muscle, where they undergo NETosis instead of releasing NETs into the circulatory system, thereby exacerbating systemic vascular inflammation. Clinical observations have shown that CitH3 levels correlate negatively with blood pressure parameters in patients with PE, suggesting that it plays a significant role in the excessive vascular inflammatory response associated with hypertension. As no abnormal changes in CitH3 levels were observed in patients with isolated gestational hypertension, the combined detection of CitH3 and PlGF may improve the early identification of individuals at high risk of PE (163).

However, studies investigating specific HPTMs as circulating biomarkers of PE remain scarce. Notably, the functions of HPTMs are highly cell type- and genomic locus-specific, and peripheral blood-based detection alone cannot capture cellular- and locus-level resolution. Moreover, HPTMs signals in peripheral blood originate from the placenta, circulating immune cells, and other tissues, making it difficult to trace them back to placental pathology with high specificity. In addition, the abundance of specific free HPTMs in circulation is extremely low, imposing high requirements for the sensitivity and specificity of detection technologies.

Given the difficulties associated with the direct detection of HPTMs, researchers have progressively shifted their focus to the enzymes that catalyze these HPTMs. Generally, these enzymes are more stable within cells than their catalytic products, and changes in their expression levels may be more easily detected in circulation, thereby offering more feasible indirect indicators for diagnosis. Previous studies have shown that JMJD6 expression is downregulated in placenta-derived sEVs from 22 weeks of gestation to delivery and that its diagnostic accuracy for EOPE (AUC = 0.828) is higher than that for LOPE (AUC = 0.704). Importantly, the downregulation of JMJD6 is accompanied by an aberrant increase in fibronectin (FN) levels in circulating placental sEVs from patients with EOPE. In addition, the combined FN^+^/JMJD6^-^ signature significantly improved the predictive value for EOPE, with the area under the curve (AUC) increasing to 0.980 (167). Mechanistically, JMJD6 inactivation induces aberrant glycosylation and lysosomal accumulation of FN under severe placental hypoxia, impairing EVT invasion of the uterine spiral arteries. Restoration of JMJD6 activity through Fe²^+^ supplementation reversed this pathological process (183). However, it should be noted that these data were derived from small, single-center cohorts without independent validation, and incremental value over established markers such as sFlt-1/PlGF has not been demonstrated. High AUC values obtained under such conditions should not be interpreted as evidence of clinical implementation. Xu et al. found that JMJD3 mRNA levels were significantly elevated in the peripheral blood mononuclear cells of patients with PE, which may indirectly reflect the disease-associated systemic inflammatory immune state, serving as a potential biomarker for evaluating immune imbalance (110). However, this study did not stratify the enrolled participants into EOPE and LOPE subgroups. Given the distinct epigenetic backgrounds underlying EOPE and LOPE, the HPTM profiles in placental tissues and maternal circulation may differ substantially between PE subtypes. Overall, this underscores the importance of emphasizing patient population homogeneity when investigating HPTMs as non-invasive biomarkers for PE.

## 6 Conclusion and perspectives

Existing reviews on epigenetics in PE have discussed HPTMs largely as a secondary topic alongside DNA methylation and non-coding RNAs, and some authors have explicitly noted that studies investigating the role of HPTMs in PE remain limited. These reviews are confined to histone methylation and acetylation, with emerging HPTMs left unaddressed (184, 185). They generally do not distinguish the sources of evidence, presenting findings from human placental tissues, animal models, and trophoblast cell lines without differentiation. Moreover, discussions of HPTM regulatory mechanisms frequently neglect the distinct placental pathologies of EOPE and LOPE.

In addition to histone methylation and acetylation, this review integrates emerging HPTMs, lactylation, ubiquitination, and citrullination, and provides a relatively comprehensive account of their potential regulatory roles in PE pathogenesis, including trophoblast differentiation and function, placental structural remodeling, microenvironmental dysregulation, and the resulting immune imbalance at the maternal-fetal interface and maternal endothelial dysfunction. In examining the mechanisms by which specific histone-modifying enzymes and their catalytic products may participate in PE pathology through downstream gene networks, we emphasized the distinction of evidence by source and considered the pathological differences between the two PE subtypes. Based on these findings, we discuss the potential of these epigenetic regulatory factors as candidate therapeutic targets and diagnostic biomarkers.

Importantly, the remarkable complexity of HPTMs in the pathogenesis of PE emphasizes that investigations into specific modifications must integrate analyses of their cellular localization, target gene context, and pathological stage. For example, the abnormal enrichment of H3K4me3 in specific gene promoter regions of CTBs has been reported to inhibit cell fusion. In contrast, the overall reduction in H3K4me3 in STBs reflects a decline in functional transcriptional activity, suggesting that the use of modification levels alone to determine disease status is not rigorous. Under hypoxic stress, H3K4me3 exhibits temporal dynamics characterized by an early compensatory increase followed by late redistribution, indicating that static detection may not sufficiently capture its dynamic regulatory processes. During PE, the placenta undergoes stress, adaptation, and decompensation, which affect its HPTM profile. Consequently, the modification levels observed in cross-sectional studies may represent a combination of signals from various pathological processes. Placental tissue obtained at delivery represents the terminal stage of the disease, and since delivery is a vigorous physiological process that induces inflammation and hypoxia, these factors may lead to changes in HPTMs, resulting in a discrepancy between HPTM levels in samples collected at delivery and those observed during the onset and progression of PE. In addition, EOPE placentas are typically delivered earlier than LOPE placentas and full-term control placentas, making it difficult in studies without gestational age-matched controls to rule out the possibility that changes in HPTMs merely reflect differences in placental maturity. Therefore, the association between HPTMs and PE observed in cross-sectional studies should be interpreted as correlations rather than causations.

Given the distinct pathological bases, EOPE and LOPE may follow different epigenetic regulatory patterns during their development and progression. In summarizing the evidence regarding differences in HPTMs between EOPE and LOPE, we found that the available studies remain insufficient. Most mechanistic investigations of HPTMs in PE have been conducted in groups with unspecified subtypes, and only a minority of studies have clarified the PE subtype of the enrolled patients, with EOPE receiving considerably more research attention than LOPE. Among the available studies, certain HPTM dysregulation exhibits subtype specificity:: for example, reduced JMJD6 demethylase activity toward H4R3me2 has been observed exclusively in EOPE placentas, whereas global H3K27me3 reduction with compensatory activation of BMP signaling has been documented in placentas of both subtypes. Consequently, findings from PE cohorts without subtype stratification may obscure modification changes that occur in opposite directions between subtypes. Moreover, if HPTM changes observed in cohorts predominantly comprising a single subtype are generalized to the other subtype, this may not only affect the accuracy of mechanistic interpretation but also undermine the assessment of subtype applicability for their use as biomarkers or therapeutic targets. Future studies should report subtype-specific findings based on gestational age-matched controls and incorporate both the EOPE and LOPE cohorts into a single study design to allow for direct comparison.

The complexity of these mechanistic studies has extended to the development of diagnostic biomarkers. Although the detection of histone-modifying enzymes or characteristic H3 analysis of NETs based on placenta-derived sEVs has demonstrated diagnostic potential, their clinical translation remains challenging. The sFlt-1/PlGF ratio is among the more extensively evaluated biomarkers for PE and has been incorporated into clinical guidelines in several regions; however, its diagnostic efficacy and clinical utility in improving pregnancy outcomes remains debated (186–188). By comparison, the evidence supporting HPTMs-related biomarkers remains preliminary. Biomarker studies should first clarify the tissue and cellular origins of the signals, rather than merely focusing on changes in total abundance. As the most promising approach involving placental sEVs, sorting strategies based on placenta-specific surface antigens should be optimized to obtain highly purified placental sEVs populations, thereby minimizing interference from the maternal background. In addition, integrating single-cell sequencing with spatial omics technologies, such as MALDI-MS imaging, will enable *in situ* mapping of the spatial distribution of HPTMs in the placenta of patients with PE, facilitating the identification of key cell subtypes contributing to aberrant signals and the selection of targets with the highest diagnostic specificity (189). Given the dynamic nature of HPTMs, large-scale longitudinal cohorts encompassing normotensive and PE pregnancies are needed to establish baseline physiological fluctuations, thereby enabling accurate delineation of pathological deviations. In addition, the construction of molecular combinatorial diagnostic models that integrate multidimensional epigenetic information holds promise for overcoming the limitations of single biomarkers and improving the accuracy of PE subtype discrimination and disease progression monitoring.

The complexity of HPTMs in PE pathogenesis is further reflected in their interactions with other epigenetic mechanisms. In cultured trophoblast cell model, downregulation of G9a and DNMT1 jointly induces excessive trophoblast autophagy, suggesting a link between histone methylation and DNA methylation. Histone methylation and m^6^A RNA modification have been proposed to synergistically trigger trophoblast ferroptosis through the MLL1–RBM15–m^6^A axis, while direct evidence in PE placentas remains limited. Furthermore, the inhibition of trophoblast cell invasion by H3K4me3 via upregulation of LncRNA-HEIPP, together with the indirect regulation of EMT by HDAC4 through miRNAs, suggests a possible link between HPTMs and non-coding RNA regulation. However, these interactions require validation in PE patient-derived tissues or cells. In addition, pathological processes such as hypoxia, oxidative stress, inflammation, and metabolic reprogramming coexist in PE, resulting differences in HPTMs are the outcome of the synergistic effects of multiple pathological stimuli, rather than the result of a single factor.

Despite the complexity of regulatory networks, certain molecules function as multidimensional hubs that connect distinct pathological processes. For instance, aberrant upregulation of MLL1 regulates syncytialization through *TEAD4* and promotes trophoblast ferroptosis via the RBM15–m^6^A axis. Reduced JMJD6 activity simultaneously impairs placental hypoxic response and trophoblast invasion. Overall, these multifunctional regulatory nodes represent promising candidate targets for PE intervention. The multifunctionality also suggests that preclinical evaluations should comprehensively consider the multiple effects that targeting a particular molecule may produce, rather than focusing on a single signaling pathway. Epigenetic drugs targeting these nodes have shown potential for ameliorating placental pathology in preclinical studies. However, their safety, effective dosage, and long-term effects on fetal development in patients with PE remain unclear. Therefore, systematic and standardized preclinical studies are essential for evaluating the clinical value and risks associated with pharmacological interventions. Placenta-targeted drug delivery may represent a promising investigational strategy with the potential to reduce interference with other maternal tissues and fetal developmental epigenetic programming. Moreover, any intervention in the epigenetic state at the maternal-fetal interface must be accompanied by a comprehensive assessment of the potential risks to fetal genomic imprinting, developmental programming, and long-term health. Given the high difficulty and risks associated with the development of new targeted drugs, repurposing existing drugs has emerged as a more realistic and feasible translational approach to drug development. For example, metformin, P4, and melatonin have been found to ameliorate trophoblast inflammation and dysfunction by modulating HPTM-related pathways, such as the ATXN7L3/H2BK120ub and SETD7/HTRA1 axes, providing new epigenetic evidence supporting their use in the prevention and treatment of PE.

In conclusion, exploring the mechanistic roles of HPTMs in the onset and progression of PE may provide new perspectives for understanding the therapeutic effects of existing drugs and an important foundation for developing more targeted diagnostic and therapeutic strategies based on epigenetic regulation.
